# Within- and between-day associations between children’s sitting and physical activity time

**DOI:** 10.1186/s12889-015-2291-3

**Published:** 2015-09-23

**Authors:** Nicola D. Ridgers, Anna Timperio, Ester Cerin, Jo Salmon

**Affiliations:** Centre for Physical Activity and Nutrition Research, Deakin University, 221 Burwood Highway, Burwood, VIC 3125 Australia

**Keywords:** Activitystat hypothesis, Accelerometry, Sedentary behaviour, Youth

## Abstract

**Background:**

The objective of this study was to examine whether increased levels of sitting time and physical activity in one period (within-day) or on one day (between-day) were predictive of lower levels in these behaviours in the following period or day among children.

**Methods:**

Children aged 8–11 years from 8 primary schools located in Melbourne, Australia, wore an activPAL for 7 consecutive days (*n* = 235; 53 % boys). Sitting, standing and stepping time were derived for each day and for specific periods on weekdays and weekend days. Multilevel analyses were conducted using generalised linear latent and mixed models to estimate associations between temporally adjacent values (i.e. pairs of days; pairs of periods within-days) between the outcome variables.

**Results:**

Significant associations were observed between temporally adjacent days and periods of the day. On any given day, an additional 10 min of stepping was associated with fewer minutes of stepping (~9 min; 95 % CI: −11.5 to −6.2 min) and standing (15 min; 95 % CI: −18.8 to −11.1 min) the following day. Greater time spent sitting during one period, regardless of being a weekday or weekend day, was associated with less time sitting and more time standing and stepping in the following period.

**Conclusions:**

The direction of the results suggest that children appeared to compensate for increased sitting, standing, and stepping time both within- and between-days. The implications of such associations for the design and delivery of interventions require consideration.

## Background

The importance of regular physical activity participation for children’s physical, social, mental and emotional health has been well documented [1]. In recent years, research has also investigated the health consequences of sedentary behaviour. Whilst most of the health evidence relating to children’s sedentary behaviour has been limited by the use of cross-sectional studies, self-report measures, and a lack of adjustment for potential confounders [2], the effect of behaviours such as television viewing and sitting time on obesity and cardiovascular disease risk factors, independent of physical activity, have also been reported [2, 3]. The recently updated Australian government guidelines for physical activity and sedentary behaviour recommend that children should accumulate at least sixty minutes of moderate- to vigorous-intensity physical activity (MVPA) every day and limit their use of electronic entertainment media to no more than two hours a day [4]. However, less than 10 % of Australian children aged 9–11 years currently meet both the physical activity and sedentary behaviour recommendations, respectively [5]. Low levels of physical activity and high levels of screen-time have also been reported in the United States and United Kingdom, for example [6, 7]. Consequently, the development and implementation of strategies to increase children’s physical activity and reduce sedentary behaviour are population health priorities.

One potential challenge to increasing physical activity and reducing sedentary behaviour is a hypothesised innate activity set-point (termed the ‘activitystat’) [8]. The ‘activitystat’ hypothesis suggests that children compensate for increased physical activity at one time point by reducing their physical activity at another time point, thus maintaining their total activity set-point [8]. Compensation has potential implications for the promotion of physical activity and the reduction of sedentary behaviour [9, 10]. For example, an intervention designed to promote active transport to school may need to be supplemented with additional promotion of activity throughout the school day to prevent compensation as hypothesised by the activitystat hypothesis. Due to potential compensation across postures, strategies to reduce sitting may also be an important focus of physical activity interventions. Objective techniques for measuring physical activity/movement, such as accelerometers, provide ideal opportunities to explore compensation as data are date and time-stamped [11], enabling exploration of associations between adjacent periods of the day (within-days) and adjacent days (between-days).

To date, observational and experimental research investigating activity compensation using accelerometry have reported inconsistent findings but have been hampered by methodological shortcomings [12]. Using between-person designs, several studies have reported that youth with varying opportunities for physical activity engagement during the school day engage in similar volumes of total daily physical activity [13, 14]. However, between-person designs cannot approximate the within-person nature of an activity set-point. In contrast, studies using within-person designs have largely found that youth did not compensate their physical activity levels between [15, 16] or within [17–19] days but typically have not accounted for ‘person-level activity’ (mean activity level of the individual), around which activity may fluctuate. A recent study that did adjust for individual differences in mean activity levels found temporal associations between pairs of days, suggesting that children appeared to compensate for increases in activity on one day by decreasing their activity levels the following day [20].

To date, the majority of research on physical activity compensation has focused solely on MVPA, though changes along the activity spectrum (i.e. sedentary, light, MVPA) are plausible in response to active or sedentary behaviours [8]. That is, increased physical activity (regardless of intensity) or sedentary time during one period may result in decreased physical activity or sedentary time in a subsequent period to maintain an innate set-point [8]. In one of the only studies to examine compensatory changes on different intensities of activity, Ridgers et al. found negative associations between accelerometer-determined sedentary time on any given day and sedentary time the following day, and for light-intensity physical activity (LPA) with subsequent LPA and MVPA [20]. A limitation of accelerometers, however, is that sedentary time is estimated based on lack of movement, rather than the way in which a sedentary behaviour may be manifested (reclining or sitting posture) [21]. Furthermore, light-intensity activities, such as standing, which register little or no movement on hip-mounted accelerometers yet require a greater energy expenditure compared to sitting/reclining postures, may be misclassified using accelerometry [22].

A recent study reported that mean week-to-week differences in the proportion of time that children spend in different postures was small [23]. To the best of our knowledge, however, no research has specifically examined correlations between the amount of time children spend in different postures (e.g., sitting, standing, stepping) either within- or between-days. The aim of this study, therefore, was to examine temporal correlations between primary school children’s sitting, standing and stepping time, estimated using the activPAL within and between days. Given the current research agenda to develop efficacious strategies to increase physical activity and reduce sitting time among children [24], establishing whether such temporal correlations occur will contribute to the design and delivery of such interventions.

## Methods

### Participants

Data were drawn from the baseline assessment (August-September 2012) of the Patterns of Habitual Activity across Seasons (PHASE) Study [25]. Primary schools located within a 40 km radius of the Melbourne Central Business District and with an enrolment of ≥200 pupils were stratified into tertiles of socioeconomic status (SES) based on postcode using the Socio-Economic Index for Areas [26]. Schools were randomly selected from each SES stratum and invited to participate in the study. Nine schools (1 low SES, 3 medium SES, and 5 high SES) agreed to participate and returned informed consent from the School Principal. All children in Years 4 and 5 (aged 8–11 years) were invited to participate in the study (*n* = 1270). Informed written parental consent was provided for 326 children (162 boys, 164 girls; 26 % response rate) to participate in at least one component of the study at baseline [25]. Ethical approvals for the study were provided by the Deakin University Human Ethics Advisory Group (Health), the Department of Education and Early Childhood Development, and the Catholic Education Office (Melbourne).

### Measures

*activPAL:* A subsample of children (*n* = 235; 125 boys, 110 girls) from 8 schools had their sitting/lying (subsequently referred to as sitting), standing and stepping time measured for 8 consecutive days using an activPAL (PAL Technologies Ltd, Glasgow, UK). The activPAL is a small accelerometer worn midline on the anterior aspect of the thigh that detects limb position, and has acceptable validity for measuring primary school children’s sitting, standing and stepping time [27]. The monitor was enclosed in a small pocket on an adjustable elasticised belt. Data concerning limb position were sampled at 10 Hz. Children were instructed to wear the monitor during all waking hours except during water-based activities, and were provided with information concerning the correct wear and care of the monitor.

#### Anthropometry

Measurements of stature (to the nearest 0.1 cm) and body mass (to the nearest 0.1 kg) were recorded using a portable stadiometer (Model 217; SECA, Germany) and calibrated electronic scale (Tanita BC-351; Tanita, Japan), respectively. Waist circumference (to the nearest mm) was measured using a flexible steel tape at the narrowest point between the bottom rib and the iliac crest, in the midaxillary plane. Each child’s body mass index (BMI; kg/m^2^) was also calculated. All measurements were taken using standardised techniques [28].

#### Data reduction

Time and date-stamped activPAL data were downloaded in 15-s epochs using manufacturer proprietary software (activPAL Professional v7.2.28) and processed using a customised Excel macro. The time that children spent sitting, standing, and stepping during each measured day and within specific periods on weekdays (before school, during school, and after school based on bell-times provided by each school) and weekend days [morning (6–12 pm), afternoon (12–6 pm), evening (6–10 pm)] were derived. To the best of our knowledge, there are no published activPAL non-wear criteria, therefore 20 min of consecutive zero accelerometer counts was used to identify periods of non-wear. This definition is commonly used in accelerometry studies with children of this age [29]. A valid day was defined as ≥8 h of wear time on weekdays and ≥7 h on weekend days [30]. For the purposes of the between- and within-day analyses, three different inclusion criteria were used. To be included in the between-day analyses, children were required to have worn the monitor for a minimum of any three days. Two hundred children (105 boys, 95 girls) met this criterion. For inclusion in the within-weekday analyses, children were required to have ≥3 valid week days (88 boys, 86 girls provided data). Two valid weekend days were required for the weekend day analyses (50 boys, 53 girls provided data). These within-day criteria were used as different patterns of physical activity and sedentary time have been observed within weekdays and weekend days [31, 32].

#### Statistical analyses

Descriptive analyses were initially calculated for all measured variables. To account for the hierarchical nature of the collected data, multilevel analyses were performed using the generalised linear latent and mixed models (GLLAMM) procedure in Stata SE version 12 (StataCorp LP, College Station, Texas). Multilevel models are the most appropriate technique for analysing hierarchical, correlated data [33]. Initial GLLAMMs were conducted to examine differences in descriptive data and activPAL outcomes between children with complete and incomplete activPAL data, and those who were included in both within-day analyses versus those who were included in either the within-weekday or within-weekend day analyses, adjusting for clustering at the school level.

The main analysis consisted of GLLAMMs, which estimated temporal correlations between adjacent values (i.e. pairs of days; pairs of periods within-days) between the outcome variables whilst adjusting for person-level (overall mean daily) sitting, standing or stepping time as appropriate [20]. These analyses examined whether the amount of time a child spent in an identified posture (e.g., sitting) during any given day (between-day analyses; day *d*) or period (within-day analyses; period *p*) was correlated with the time spent in that posture during a previous day (day *d-1*) or period (period *p-1*), respectively. As data were collected for eight consecutive days, each child provided seven data points for the between-day analyses (e.g. day 2 (*d*) compared with day d (*d-1*) etc.), and ten and four data points (e.g. e.g. P3 (*p*) compared to P2 (*p-1*), P2 (*p*) compared to P1 (*p-1*) on each day) for the within-weekday and weekend day analyses, respectively. In total, 826 data points were analysed for the between-day analyses, and 1524 and 367 data points for the within - week day and weekend day analyses, respectively. All models were specified to have random intercepts at the school, person and day levels, and random slopes at the person level for sitting, standing and stepping time variables at *d-1* or *p-1*. The GLLAMMs were also adjusted for sex, year of school (Year 4 or Year 5), day of measurement, waist circumference, and activPAL wear time. The significance level was set at *p* < 0.05.

## Results

On average, children were 10.1 (±0.7) years old, had a BMI of 18.8 (±3.5) kg/m^2^ and a waist circumference of 69.4 (±10.7) cm. No significant differences between children included and excluded from the between-day and within-day analyses were observed for any of the descriptive variables (*p* > 0.05). No significant differences in the demographic and activPAL data were observed between children who provided data for both the within-weekday and within-weekend day analyses compared to those children only included in one within-day analysis, though children included in both analyses had significantly lower BMI (0.99 kg/m^2^; *p* = 0.034) and significantly higher average day wear time (123.9 min; *p* < 0.001). Table 1 summarises activPAL data for the between-day and within-day samples.

**Table 1:** Descriptive statistics of activPAL data for an average day, and weekday and weekend day periods (hrs/day; mean ± SD)

	Average day (*n* = 200)	Weekday (*n* = 174)			Weekend (*n* = 103)		
		Before school	At school	After school	Morning	Afternoon	Evening
					(6 am-12 pm)	(12-6 pm)	(6-10 pm)
Sitting time (hrs/day)	8.7 (2.7)	1.1 (0.5)	3.5 (0.5)	3.2 (0.9)	2.6 (1.2)	3.3 (0.9)	2.3 (0.7)
Standing time (hrs/day)	3.2 (0.8)	0.4 (0.2)	1.5 (0.4)	1.2 (0.4)	0.8 (0.5)	1.4 (0.6)	0.6 (0.4)
Stepping time (hrs/day)	2.0 (0.5)	0.3 (0.1)	1.1 (0.2)	0.8 (0.3)	0.5 (0.3)	0.9 (0.5)	0.3 (0.2)
Wear time (hrs/day)	13.8 (2.9)	1.8 (0.6)	6.1 (0.3)	5.1 (1.1)	3.9 (1.3)	5.7 (0.7)	3.2 (0.8)

The temporal correlations between time spent sitting, standing and stepping on adjacent days are presented in Table 2 (between-day analyses). Six statistically significant negative associations were observed between temporally adjacent values on the outcome variables. Time spent sitting, standing and stepping on any given day were each temporally correlated with less time spent standing and stepping, but not sitting, the following day. For example, an additional 10 min in stepping time on any given day was temporally correlated with fewer minutes of stepping (8.8 min; *p* < 0.001) and standing (15 min; *p* < 0.001) the following day. The smallest yet significant negative between-day temporal correlations were observed for sitting time on one day and standing and stepping time the following day.

**Table 2:** Associations of time (min) children spent in different postures between pairs of days (significant results in bold)

	Between-days model^a^	
	b (95 % CI)	*p* value
Sit_D1_ → Sit_D2_	−0.02 (−0.08 to 0.03)	0.444
Stand_D1_ → Stand_D2_	**−0.78 (−0.98 to −0.58)**	<0.001
Step_D1_ → Step_D2_	**−0.88 (−1.15 to −0.62)**	<0.001
Sit_D1_ → Stand_D2_	**−0.05 (−0.09 to −0.02)**	0.001
Sit_D1_ → Step_D2_	**−0.02 (−0.05 to −0.01)**	0.036
Stand_D1_ → Sit_D2_	0.34 (−0.01 to 0.69)	0.055
Stand_D1_ → Step_D2_	−**0.46 (−0.60 to −0.32)**	<0.001
Step_D1_ → Sit_D2_	−0.29 (0.98 to 0.41)	0.416
Step_D1_ → Stand_D2_	**−1.50 (−1.88 to −1.11)**	<0.001

Abbreviations: Sit = Sitting time; Stand = Standing time; Step = Stepping time; b = point estimate of the regression coefficient; 95 % CI = 95 % confidence intervals; D = day

^a^Model: Adjusted for sex, year of school, day of measurement (e.g. Monday, Tuesday, etc.), waist circumference, wear time on a given day, and average person-level activity and/or sitting time (as appropriate) per day

Note: b indicates the association between days for every additional minute of activity on one day and activity the following day

The temporal correlations between time spent sitting, standing and stepping in adjacent periods of the day on weekdays and weekend days are presented in Table 3 (within-day analyses). All temporal correlations were significant (all *p* < 0.01); the exception being standing in one weekday period and stepping in the following period. The direction of the significant temporal correlations between periods was the same for both weekdays and weekends. For example, during any given period on any given weekday, an additional 10 min in sitting time was associated with fewer minutes of sitting (3.7 min; *p* < 0.001), and more minutes of standing (0.6 min; *p* = 0.004) and stepping (1.9 min; *p* < 0.001) in the following period. Similarly, on weekend days, an additional 10 min of sitting time in one period was associated with fewer minutes of sitting time (2.9 min), and 3.7 and 3.0 more minutes of standing (3.7 min) and stepping (3.0 min) time, respectively, in the following period (all *p* < 0.001).

**Table 3:** Associations of time (min) children spent in different postures between periods within weekdays and weekend days (significant results in bold)

	Weekdays^a^		Weekend days^a^	
	b (95 % CI)	*p* value	b (95 % CI)	*p* value
Sit_P1_ → Sit_P2_	**−0.37 (−0.43 to −0.30)**	<0.001	**−0.29 (−0.39 to −0.19)**	<0.001
Stand_P1_ → Stand_P2_	**−0.40 (−0.45 to −0.35)**	<0.001	**−0.51 (−0.60 to −0.42)**	<0.001
Step_P1_ → Step_P2_	**−0.48 (−0.53 to −0.42)**	<0.001	**−0.68 (−0.76 to −0.60)**	<0.001
Sit_P1_ → Stand_P2_	**0.06 (0.02 to 0.11)**	0.004	**0.37 (0.32 to 0.42)**	<0.001
Sit_P1_ → Step_P2_	**0.19 (0.17 to 0.22)**	<0.001	**0.30 (0.25 to 0.35)**	<0.001
Stand_P1_ → Sit_P2_	**0.82 (0.76 to 0.89)**	<0.001	**0.93 (0.78 to 1.07)**	<0.001
Stand_P1_ → Step_P2_	0.02 (−0.02 to 0.05)	0.389	**−0.17 (−0.26 to −0.09)**	<0.001
Step_P1_ → Sit_P2_	**1.24 (1.15 to 1.35)**	<0.001	**1.2 (1.03 to 1.38)**	<0.001
Step_P1_ → Stand_P2_	**−0.14 (−0.23 to −0.05)**	0.002	**−0.34 (−0.46 to −0.21)**	<0.001

Abbreviations: Sit = Sitting time; Stand = Standing time; Step = Stepping time; b = point estimate of the regression coefficient; 95 % CI = 95 % confidence intervals; P = within-day period

^a^Model: Adjusted for sex, year of school, day of measurement (e.g. Monday, Tuesday, etc.), waist circumference, wear time in a given period, and average person-level activity and/or sitting time (as appropriate) per period and per day

Note: b indicates the association within-days for every additional minute of activity in one period and activity in the following period

## Discussion

This study used a within-person design to examine whether increased levels of sitting, standing and stepping time in one period (within-day) or on one day (between-day) were temporally correlated with lower levels in these behaviours in the following period or day among children. The direction of the results are consistent with those predicted by the activitystat hypothesis, whereby higher levels of activity in one period or on one day were temporally correlated with lower levels of activity in the following period or day. Notably, apparent compensatory changes around which activity would be hypothesised to fluctuate if each individual did have an activity set-point [8] were observed once person-level mean sitting, standing and/or stepping time were included in the analyses.

Little is currently known about the temporal characteristics of children’s activity patterns [15, 34]. It has been suggested that activity compensation, as predicted by the activitystat hypothesis, is unlikely to be observed within-days [12], which has been supported by studies demonstrating no evidence of within-day compensatory changes in children and adolescents [15–18]. However, a variety of different approaches have been used, including examining associations between specific active behaviours [16] and the total accumulated daily physical activity and sedentary time [15] engaged in on the same day. Few studies have examined temporal correlations between periods of the day (e.g. in school, out of school) using a within-person design. Long and colleagues reported that each additional minute of MVPA engaged in during school time was associated with a 0.14 min increase in MVPA outside of school [18], while an experimental study found that children did not increase their activity levels outside of school on days where physical activity opportunities during school time were removed [17]. In contrast, in the present study we found significant negative temporal correlations between the same postures between periods, whilst significant positive and negative temporal correlations were observed between different postures across periods on weekdays and weekend days, depending on the intensity of activity investigated. It should be noted that whilst these findings are consistent with the activitystat hypothesis, it is possible that these findings may be alternatively explained by diurnal and circadian rhythms. Further research is needed to explore factors that may explain these temporal correlations observed within- and between-days in primary school-aged children.

It is possible that the contrasting results between this study and previous research are explained, in part, by the lack of adjustment for participants’ average activity levels [20]. In addition, whilst the majority of studies have used multilevel approaches to analyse data, differences in the number of days included in the analyses, valid wear time criteria, and covariates included in the models may also account for some of these differences. Overall, whilst it is acknowledged that the current study is observational in design, the results suggest that if interventions target increased physical activity or decreased sitting during specific periods of the day, regardless of whether it is a weekday or weekend day, strategies to reduce potential compensatory responses in subsequent time periods may be necessary to increase overall daily physical activity levels .

In the present study, temporal correlations between days were also explored. For standing and stepping, increased time in these activities on one day were temporally correlated with reduced time spent in these activities the following day, which is consistent with the proposed activitystat hypothesis [8]. These results contrast the findings of Baggett and colleagues [15] who reported positive associations for MVPA between pairs of days, but did not adjust for person-level activity. Interestingly, the current study found no significant temporal correlations between standing and stepping on one day and the amount of time spent sitting the following day. A previous study using accelerometry found no associations between LPA and sedentary time the following day, which is consistent with the present study, though a positive association was noted between MVPA and sedentary time the following day [20]. This latter finding may indicate that engagement in activity on one day stimulated increased activity the following day (activity synergy) [16]. Notably, the present study found no significant temporal correlations for sitting time; that is, increased sitting time on one day did not result in decreased sitting time the following day. This between-day finding lends some support to a recent experimental study that found children did not compensate their activity levels following an acute bout of sitting [35], but contrasts a recent observational study that found sedentary time compensation occurred between days [20].

Only two significant temporal correlations were observed that were not consistent with activity compensation. These were the negative temporal correlations observed between sitting time on one day and standing and stepping time the following day. Whilst the correlations observed were small, they are similar to the negative association observed between physical inactivity (sedentary time) and total accumulated physical activity between days by Baggett and colleagues, who suggested that physical activity was being displaced by inactivity [14]. Whilst is it plausible that displacement of activity by inactivity may occur within days due to the finite time people have to be active each day [15], whether this occurs between days is unclear. It is possible that lower levels of standing and stepping were observed following days of increased sitting as opportunities to increase activity levels may not have been provided [16] at school or at home, for example. A strength of the present study, however, was the adjustment for the usual activity/sitting levels of participants across the week, which would help to account for a possible individual activity set point.

This study, to the best of the authors’ knowledge, is the first to assess sitting, standing and stepping time using a validated tool and a within-person analytical approach to examine associations between activity levels within- and between- days. However, there are several limitations that warrant mention. Firstly, this study utilised an observational design. Whilst it contributes to the limited body of knowledge to date, experimental research is needed to further investigate compensation and determine whether increased periods of inactivity or activity result in compensatory changes in activity. Different time scales should be explored (e.g. within-day, between-day, between seasons) to examine whether the magnitude of the temporal correlations differ. Such research will provide information on whether an ‘activitystat’ exists and if so, how it could potentially be overridden to benefit children’s activity levels [34]. Secondly, this study examined short-term changes (i.e. within and between days). There is currently no consensus over the time frame that compensatory responses would be expected to be observed, and it has been recommended that longer time frames should also be explored [12]. Thirdly, the majority of the sample was recruited from high SES schools. Future research should examine whether factors such as SES may moderate the observed associations.

## Conclusion

Overall, the direction of the results observed suggest that an increase in sitting, standing and stepping time during one day or one period of the day were associated with a decrease in sitting, standing and stepping in the temporally adjacent day or period of the day once person-level mean sitting, standing and/or stepping times were accounted for. These findings are consistent with those proposed by the activitystat hypothesis. Experimental designs are needed to further explore such temporal correlations across the activity spectrum in order to develop targeted behavioural interventions that aim to reduce this variation in activity levels observed within and between days.
